# Development and validation of a clinico-histological factor-based nomogram for survival in sinonasal malignancies

**DOI:** 10.1038/s41598-026-41278-9

**Published:** 2026-02-25

**Authors:** Chang-Ying Zhong, Chang She, Shuang-Shuang Wang

**Affiliations:** 1 Otolaryngology Department, Hunan Provincial Hospital of Integrated Traditional Chinese and Western Medicine, No.58 Lushan Road, Yuelu District, Changsha, 410006 Hunan China; 2 Changsha Hospital of Traditional Chinese Medicine (Changsha Eighth Hospital), No.22 Xingsha Avenue, Changsha, 410100 Hunan China

**Keywords:** Nomogram, Survival-prognosis, Sinonasal malignancies, Surgery, Radiation, Chemotherapy, Head and neck cancer, Nomograms

## Abstract

The survival prognosis nomogram is needed for sinonasal malignancies with different histology types. Thus this research collected cases with sinonasal malignancies from The Surveillance, Epidemiology, and End Results (SEER) program. R was the tools for screening the predictors by Accelerated Failure Time model and “backward” elimination. Nomogram was constructed with predictors. C-index, calibration curves and decision curve analysis were applied for the validation of the nomogram. 5795 cases were collected for constructing nomogram. “Age”, “Sex”, “Marriage”, “Site”, “AJCC”, “Surg”, “Radiation”, and “Hist” were retained as predictors. The C-index was 0.77 (95% CI: 0.76–0.79, validation set). Calibration curves were close to the diagonal line. Decision curve analysis showed nonmogram provided positive net benefits relative to the default “treat-all” and “treat-none” approaches. We also conducted survival analysis for seven histology types with treatment (squamous cell carcinoma, adenocarcinoma, adenoid cystic carcinoma, undifferentiated carcinoma, primary mucosal melanoma, neuroendocrine carcinoma, olfactory neuroblastoma ). In conclusion, we constructed a nomogram to predict the survival prognosis of patients with sinonasal malignancies. It can be utilized by clinicians to predict individual survival outcomes and for patient consultation. Besides, we provided survival status for seven histology types of sinonasal malignancies with common and controversial treatment.

**Supplementary Information:**

The online version contains supplementary material available at 10.1038/s41598-026-41278-9.

## Introduction

Sinonasal malignancies are infrequent neoplastic conditions in the head and neck region. Common symptoms include nasal obstruction, epistaxis, and facial pain. In advanced cases, these tumors may cause proptosis, diplopia, and cranial neuropathy^1,2^. The incidence rate is relatively low, approximately 0.5 per 100,000 individuals^3^. The male-to-female ratio of patients with this disease is approximately 2:1^4^. In relevant studies conducted in Germany, it was found that the 5 year overall survival (OS) rate for sinonasal malignancies was 63%^5^. The 5 year OS rate for paranasal sinus malignancies was merely 52%^6^. The pathological types of sinonasal malignancies were predominantly sinonasal squamous cell carcinoma (SNSCC), followed by sinonasal adenocarcinoma (SNAC). Other pathological types were diverse but even rarer, with small sample sizes for each type. These types included sinonasal adenoid cystic carcinoma (SNACC), sinonasal undifferentiated carcinoma (SNUC), primary mucosal melanoma (PMM), sinonasal neuroendocrine carcinoma (SNEC), and olfactory neuroblastoma (ONB), among others.

While sinonasal malignancies are relatively rare, otorhinolaryngology (ENT) clinicians frequently encounter such patients with diverse histological types and varying prognoses. A primary concern for patients is treatment efficacy. Therefore, the development of a prognostic nomogram serves two key purposes. First, it offers non-clinical benefits by providing an intuitive tool that facilitates transparent communication between clinicians and patients, thereby helping to manage expectations. Second, it provides clinical benefits by serving as an evidence-based reference.

Furthermore, while existing prognostic models for sinonasal malignancies have been developed, they are predominantly limited to common subtypes such as squamous cell carcinoma and adenocarcinoma^7,8^. Given the considerable histological diversity of these malignancies, there remains a clear need for a more comprehensive prognostic model that incorporates a wider range of pathological types to improve clinical utility.

In addition, the nomogram itself has limitations in treatment selection (as it only presents individual options rather than combination regimens). Therefore, to address this gap, we also conducted Kaplan-Meier (KM) survival analyses on recommended and controversial treatment modalities for these rare pathological subtypes. This complementary approach, integrating findings from both the nomogram and KM curves, contributes additional evidence for understanding the prognostic landscape of this disease.

## Material and method

### Data source

The data was collected from The Surveillance, Epidemiology, and End Results (SEER) program, which included 17 registries, and the time span for all patient inclusion was from 2000 to 2021 (Incidence-SEER Research Data, 17 registries, Nov 2023 sub 2000 − 2021).

### Case inclusion criteria

(1) The age at diagnosis: 1–89 years old. (2) Sex: male or female. (3) The diagnostic time span (less than 1 year): We only choose patients diagnosed between 2010 and 2021. (4) The site classification: “Primary Site-Labeled”, with the site specified as “Nasal cavity and paranasal sinuses”. (5) The AJCC stage: 7th edition TNM classification for malignant tumors was selected. Only N2a and N3b stage of 8th edition TNM classification can not be accommodate to the 7th edition. We conducted the survival analysis for the 2 stages with restricted mean survival time (time = 3 year) to determined the classification. (6) Survival status: Alive or dead of other disease; Dead attribute of this carcinoma. The corresponding survival time was documented. (7) Treatment: The use of surgery, radiation, and chemotherapy was noted (yes or no/unknown). (8) Race: White, Asian, Black and Other race.

### Case exclusion criteria

duplicate samples were excluded for the ambiguous chronological order. Patients with ONB were not used in the model development, as their AJCC staging was missing not at random, and were only included for survival analysis. Additionally, patients with unknown survival time or survival status were also removed.

### Imputation methods

To avoid selection bias and maximize statistical power due to missingness in other variables, we performed multiple imputation for all non-outcome variables with missing data. The imputation was performed using the Multivariate Imputation by Chained Equations (MICE) procedure via the mice package in R, with 10 iterations and generating 5 imputed datasets. Given that all variables with missing data were categorical, the imputation method was specified as polytomous regression.​

Variables with missing values included Race, Marriage, Histology, and AJCC stage. Sensitivity analyses were conducted by comparing results from the imputed datasets with those from the complete-case analysis. The final estimates (e.g., regression coefficients, hazard or time ratios) and model development (including validation) were pooled according to Rubin’s rules.

### Method to construct nomogram

Survival endpoints and survival time were primary outcome. The nomogram was constructed using RStudio software (version 2024.4.1.748). Cases recorded between 2010 and 2021 were used to construct the nomogram following multiple imputation, in accordance with Rubin’s rules and to maximize statistical power. The initial predictors selected included “Sex”, “Age”, “Site”, “AJCC” (7th AJCC stage), “Surg” (surgery), “Chemotherapy”, “Radiation”, “Hist” (Histology), “Race”. The histology was divided into 6 types: “AC” (adenocarcinoma), “ACC” (adnoid cystic carcinoma), “SCC” (squamous carcinoma), “UC” (undifferentiated carcinoma), “PMM” (primary mucosal melanoma), and “Other”. The histology types of “Other” were recorded in Table S1. Continuous variables were expressed as median with inter quartile range (IQR), and categorical variables were expressed as number and percentage of cases. Variable selection was performed using a stability selection approach within the multiple imputation framework. Stepwise regression (direction: backward) based on the Bayesian Information Criterion (BIC) was conducted separately on each of the five imputed datasets, and the frequency of variable selection was recorded. Variables that were consistently selected in at least 60% of the datasets (= > 3/5) were included in the final model. All categorical variables were treated as factors.

We used Cox regression when the proportional hazards assumption held, and an Accelerated Failure Time (AFT) model otherwise. In the AFT model, effects are expressed as time ratios (TR): TR > 1 indicates longer survival, TR < 1 indicates shorter survival. In the Cox regression effect were expressed as hazard ratio (HR): HR < 1 indicates longer survival, HR > 1 indicates shorter survival. A nomogram predicting 1, 3, and 5-year survival was constructed using the rms package.

### validation of the nomogram

The data were randomly divided into a training set (70%) and a validation set (30%) for model validation, with the random seed ‘4904’. The model was fitted on the training set, and its performance was evaluated on the corresponding validation set. Three performance metrics were as bellow:

First, the concordance index (C-index) was calculated to determine the consistency between the predicted probabilities of the nomogram and the actual outcomes. A C-index value close to 1 indicates stronger predictive ability of the nomogram. In contrast, a C-index close to 0.5 suggests that the nomogram’s predictive performance is no better than random guessing. We performed 5-fold cross-validation to assess the model’s generalizability.

Second, calibration curves were employed to assess the agreement between the nomogram-predicted probabilities and the actual observed probabilities. The calibration performance was validated using an internal bootstrap resampling approach with 1,000 iterations and 10 probability bins. For each time point (1, 3, and 5 years), the following procedure was performed:

A bootstrap sample comprising 70% of the original dataset (training set) was drawn with replacement, leaving the remaining 30% as the validation set. The model was refitted on the bootstrap sample. Calibration performance, including slope and intercept, was evaluated on both the bootstrap sample (apparent performance) and the original dataset (test performance).

Third, we assessed the clinical utility of the nomogram using decision curve analysis (DCA). The net benefit of the nomogram was compared against the default strategies of treating all patients or treating none. The results depended on if the nomogram provided a higher net benefit. Additionally, another DCA based on the AJCC stage was constructed for comparison to assess whether the nomogram offered better clinical utility.

### Survival analysis

To better understand the treatment response and survival outcomes across different histology subtypes, Kaplan-Meier survival analyses were performed for seven histology types that were either commonly encountered or associated with therapeutic controversy: SNSCC, SNAC, SNACC, SNUC, SNEC, PMM, and ONB. The analysis was carried out on the complete data. Accordingly, sensitivity analyses were applied to both the imputed and the complete datasets (log-rank test).

### Statistical analysis

All variables must undergo collinearity testing, and those with a Variance Inflation Factor (VIF) greater than 5 should be excluded. For the results of regression analyses and log-rank test, statistical significance level was set at two-tailed *P <* 0.05.

### Ethics approval and consent to participate

Ethical approval did not require for the study as SEER is an open public database. The data about the patients has already be de-identificated. It didn’t not need for approval by institutional review committee.

## Result

### Baseline characteristics of patients with sinonasal malignancies

**Fig. 1:**
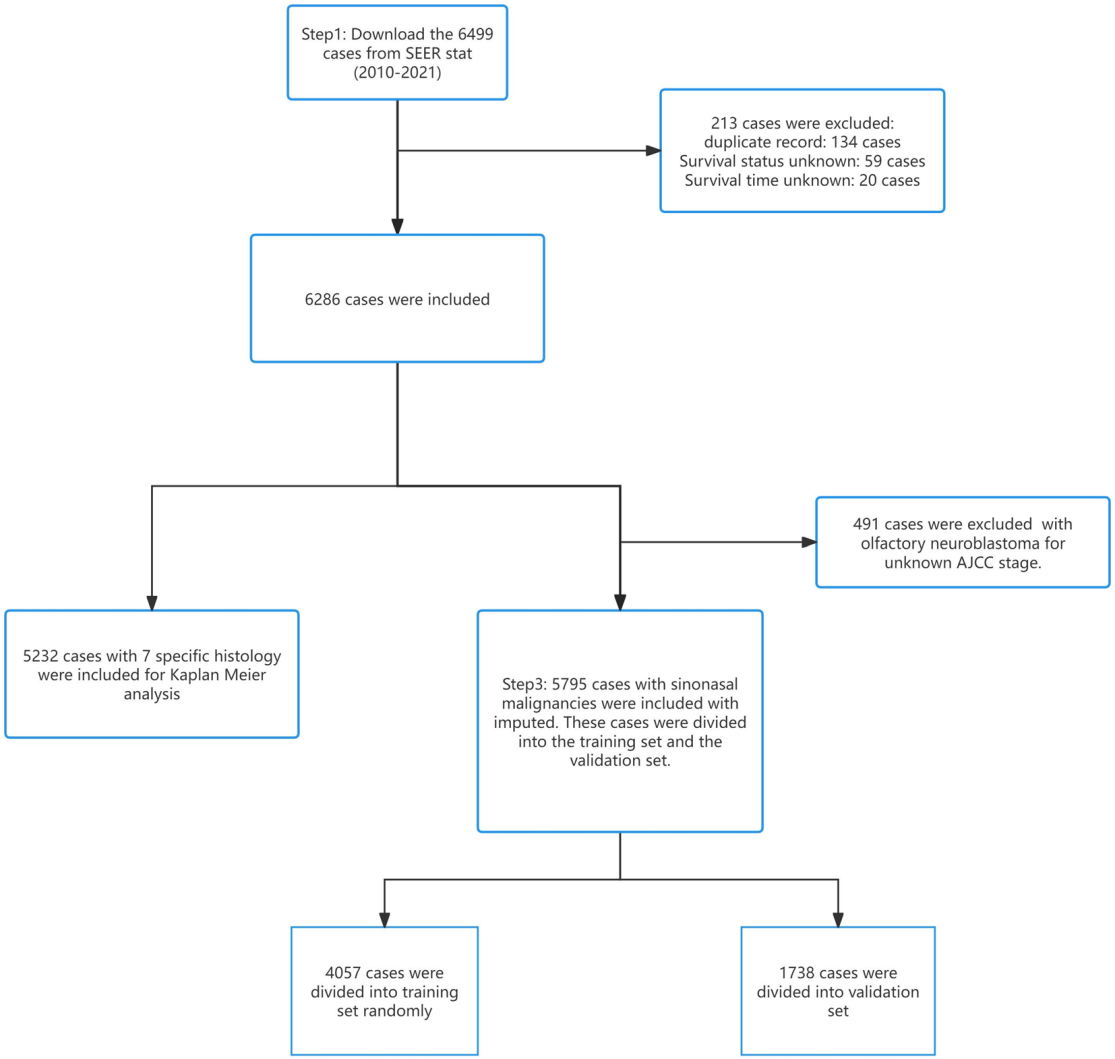
The flow chart of cases inclusion and exclusion.

A total of 6,499 cases were collected from the SEER database. As shown in Figs. 1 and 213 cases were excluded due to the duplicated records, survival status and time unknown. 6,286 cases were included.

When constructing the nomogram, 5795 patients with 6 specific histology (SCC, AC, ACC, UC, PMM and other) were involved. We also performed KM curves on the 7 common pathological subtypes including: SCC, AC, ACC, UC, ONB, PMM, and NEC.

Unexpectedly, the prognosis of 8th edition N2a was better than that of 7th edition N1 and N2, with marginal significance (*P* = 0.074 for N1, *P* = 0.033 for N2). Therefore, the mapping strategy for 8th edition N2a was reclassified to 7th edition N1. Although the difference between 8th edition N3b and 7th edition N2/N3 was not statistically significant, the mean restricted survival time of 8th edition N3b was closer to that of 7th edition N2 (difference: +0.073 years). Consequently, 8th edition N3b was reclassified to 7th edition N2.

As indicated in Table 1, The counts were presented as the average after 5 imputations, with none of the imputed variables exceeding a 5% difference in proportion. Detailed changes were in Table S2. The median age of the entire cohort was 65 years. In terms of sex distribution, males accounted for 3,573 cases (61.7%), outnumbering females (2,222 cases, 38.3%). The median survival time was 2.17 years (IQR: 0.75–5.25). Regarding marital status, married patients (3,275, 56.5%) exceeded unmarried patients (2,520, 43.5%). In terms of race, as all data were derived from the United States, White individuals constituted the vast majority (4,639, 80.1%).

**Table 1:** The baseline characteristics and regression of patients with sinonasal malignancies (imputed).

	All (*n* = 5795)	Training set (*n* = 4056)	Validation set (*n* = 1739)	All variables model		Selected variables model		
				TR(95%CI)	*P*	TR(95%CI)	*P*	Coefficient
Age Median, IQR	65 0.00 (55.00, 74.00)	65.00 (55.00, 74.60)	64.60 (54.60, 74.20)	0.976 (0.97–0.98)	< 0.001	0.973 (0.97–0.98)	< 0.001	0.02
Sex								
Female	2222 (38.3%)	1541 (38.0%)	681 (39.2%)	-	-	-	-	-
Male	3573 (61.7%)	2515 (62.0%)	1058 (60.8%)	0.84 (0.72–0.97)	0.009	0.84 (0.72–0.97)	0.019	0.18
Marriage								
Married	3275 (56.5%)	2298 (56.6%)	977 (56.2%)	-	-	-	-	-
Unmarried	2520 (43.5%)	1758 (43.4%)	762 (43.8%)	0.73 (0.62–0.85)	< 0.001	0.74 (0.63–0.86)	< 0.001	0.30
Race								
Asian	427 (7.4%)	-	-	-	-	-	-	-
Black	579 (10.0%)	-	-	1.17 (0.84–1.63)	0.345	-	-	-
Other	150 (2.6%)	-	-	1.01 (0.61–1.67)	0.965	-	-	-
White	4639 (80.1%)	-	-	1.149 (0.885–1.491)	0.296	-	-	-
AJCC stage								
I	1415 (24.4%)	984 (24.3%)	424 (24.4%)	-	-	-	-	-
II	564 (9.7%)	393 (9.7%)	163 (9.4%)	0.29 (0.20–0.41)	< 0.001	0.29 (0.20–0.41)	< 0.001	1.25
III	1042 (18.0%)	715 (17.6%)	312 (18.0%)	0.15 (0.10–0.21)	< 0.001	0.14 (0.1–0.20)	< 0.001	1.96
IVA	1293 (22.3%)	920 (22.7%)	391 (22.5%)	0.10 (0.07–0.14)	< 0.001	0.09 (0.07–0.13)	< 0.001	2.36
IVB	1099 (19.0%)	772 (19.0%)	335 (19.3%)	0.06 (0.05–0.09)	< 0.001	0.06 (0.04–0.08)	< 0.001	2.83
IVC	382 (6.6%)	272 (6.7%)	113 (6.5%)	0.03 (0.02–0.05)	< 0.001	0.03 (0.02–0.05)	< 0.001	3.46
Site								
Ethmoid sinus	473(8.2%)	323 (8.0%)	150 (8.6%)	-	-	-	-	
Maxillary sinus	1936 (33.4%)	1358 (33.5%)	578 (33.2%)	0.84 (0.65–1.08)	0.175	0.84 (0.65–1.09)	0.192	0.17
Nasal cavity	3386 (58.4%)	2375 (58.6%)	1011 (58.1%)	1.83 (1.41–2.36)	< 0.001	1.85 (1.44–2.39)	< 0.001	0.62
Histology^1^								
AC	353 (6.1%)	248 (6.1%)	108 (6.2%)	-	-	-	-	
ACC	353 (6.1%)	251 (6.2%)	101 (5.8%)	1.10 (0.70–1.74)	0.682	1.13 (0.72–1.79)	0.600	0.13
Other^2,3^	989(17.1%)	686 (16.9%)	302 (17.4%)	0.59 (0.40–0.87)	0.008	0.58 (0.39–0.86)	0.006	0.54
PMM	573(9.7%)	394 (9.7%)	179 (10.3%)	0.29 (0.19–0.45)	< 0.01	0.30 (0.20–0.46)	< 0.001	1.20
SCC	3309 (57.1%)	2320 (57.2%)	984 (56.6%)	0.66 (0.46–0.94)	0.022	0.65 (0.46–0.93)	0.018	0.43
UC	218 (3.8%)	156 (3.9%)	65 (3.7%)	0.44 (0.27–0.72)	0.001	0.42 (0.26–0.68)	< 0.001	0.87
Surgery								
No	1768 (30.6%)	1230 (30.3%)	538 (30.9%)	-	-	-	-	
Yes	4027 (69.4%)	2826 (69.7%)	1201 (69.1%)	3.17 (2.71–3.70)	< 0.001	3.26 (2.76–3.77)	< 0.001	1.17
Chemotherapy								
No	3914 (67.5%)	-	-	-	-	-	-	-
Yes	1881 (32.5%)	-	-	0.84 (0.70–1.00.70.00)	0.055	-	-	-
Radiation								
No/unknown	2399 (41.4%)	1671 (41.2%)	728 (41.9%)	-	-	-	-	
Yes	3396 (51.6%)	2385 (58.8%)	1011 (58.1%)	2.27 (1.92–2.67)	< 0.001	2.17 (1.85–2.54)	< 0.001	0.77
Survival time								
Median, IQR	2.17 (0.75, 5.25)	2.22 (0.77, 5.27)	2.18 (0.77, 5.28)	-	-	-	-	-
Status								
Alive or dead of other disease	4051 (69.9%)	4056 (69.0%)	1739 (71.5%)	-	-	-	-	-
Dead attribute of this carcinoma	1744 (30.1%)	1215 (30.0%)	529 (30.4%)	-	-	-	-	-

^1^SCC: squamous cell carcinoma; AC: adenocarcinoma; ACC: adenoid cystic carcinoma; UC: undifferentiated carcinoma; PMM: primary mucosal melanoma.

^2^During the imputation process, the limited number of NEC cases (89 cases) resulted in poor convergence of the imputed outcomes. Therefore, in constructing the model, we categorized them as “other.”.

^3^the statistical description of the imputed variables is based on the results of merging five imputed datasets using Rubin’s rule. Therefore, the total number can be accurately matched, but for example, the number of individuals at specific stages may not fully correspond, though the values are approximately equivalent.

In AJCC staging, stage IV had the highest number of patients, with 1,293cases (22.3%) in stage IVA, 1,099 cases (19.0%) in stage IVB, and 382 cases (6.6%) in stage IVC. Regarding the disease site, the nasal cavity had the highest number of cases, with 3,386 cases (58.4%). In terms of histology type, SCC was the most common, with 3,309 cases (57.1%). Regarding treatments, surgery was the most commonly used treatment, with 4,027 cases (69.4%), followed by radiation with 3,396 cases (51.6%), and chemotherapy with the lowest number of cases, 1,881 (32.5%).

### Regression and nomogram analysis

All VIF values for variables were below 5. In the proportional hazards assumption assessment, the Schoenfeld residual test *P*-value was less than 0.001. Therefore, the model was constructed based on an AFT model. We first compared the TR of all variables between the imputed dataset and the complete dataset. After imputation, only two variables showed directional changes in *P* values.

One was the “Other” category in the pathological type (Hist Other): after multiple imputation, its statistical significance markedly increased (*P*-value decreased from 0.066 to 0.008), while the direction of TR remained consistent (< 1), indicating that imputation enhanced the statistical power of this variable.

The other variable was chemotherapy, where the direction of the TR did not change, but its significance shifted from non-significant to borderline significance. Detailed comparison results are provided in Table S3.

Chemotherapy, sex and race weren’t consistently selected and included in the prognostic model after backward elimination. Sex was also forcibly retained in the model based on prior knowledge and statistical significance^9^. Chemotherapy and race were not incorporated into the final model. Thus, the final model formula was defined as:

$${\text{formula }}={\text{ Surv}}\left( {{\text{time, status}}} \right){\text{ }}\sim {\text{ Age }}+{\text{ Sex }}+{\text{ Marriage }}+{\text{ Hist }}+{\text{ Site }}+{\text{ AJCC }}+{\text{ Surgery }}+{\text{ Radiation}}$$

**Fig. 2:**
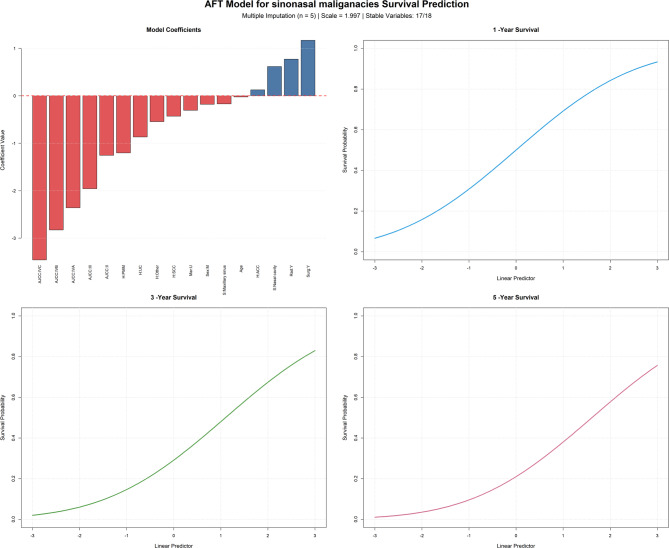
The nomogram for predicting 1, 3, and 5- year survival of sinonasal malignancies.

The pooled coefficients were shown in Table 1. It was noted that the coefficient for the hist ACC variable exhibited considerable variability (37.1%), suggesting that its estimate may be relatively unstable. Visualization of the model should be interpreted in conjunction with Table 1; Fig. 2.

The linear predictor for each patient is obtained by summing the products of variable values and their corresponding coefficients (e.g., age multiplied by its coefficient, plus fixed coefficients for staging and other categorical variables). Based on the linear predictor, the 1, 3, and 5-year survival probabilities can be read from the respective y-coordinates in the nomogram.

### Validation of the nomogram

**Fig. 3:**
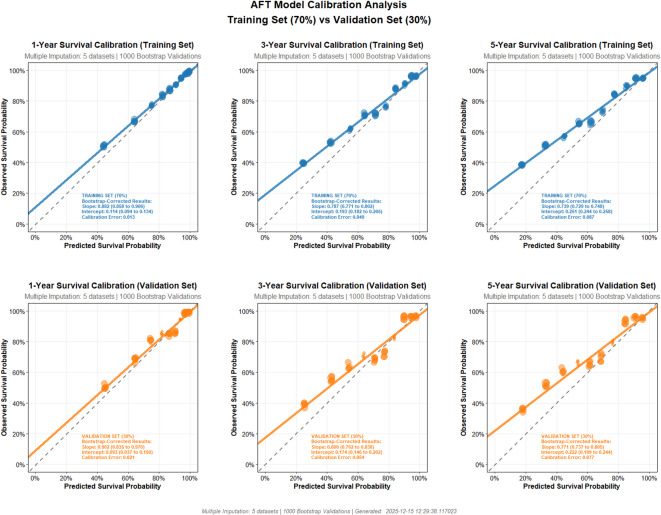
The calibration curves.

The 5-fold cross-validation yielded a C-index of 0.78 (95% CI: 0.77–0.79) for the training set and 0.77 (95% CI: 0.76–0.79) for the validation set. Optimism was only 0.004, indicating no substantial overfitting. As shown in Fig. 3, both the training and validation sets exhibited good performance in predicting 1-year survival, with slopes exceeding 0.88. In contrast, for 3-year and 5-year survival predictions, the slopes ranged between 0.70 and 0.80. The calibration curves showed slight deviation in the lower range of predicted survival probabilities but remained close to the diagonal in the higher range.

**Fig. 4:**
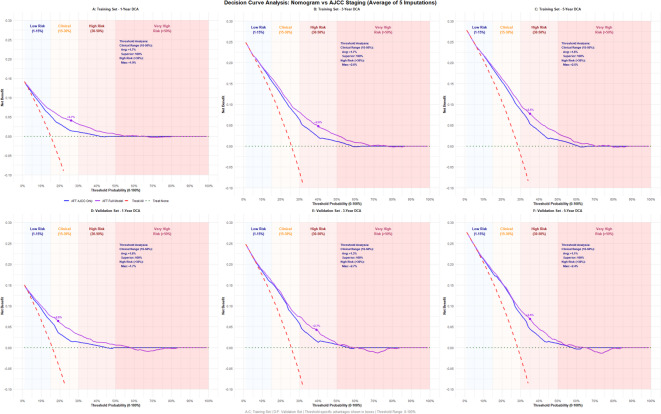
Decision curve analysis.

As can be seen from Fig. 4, DCA analysis revealed that the nomogram and AJCC 7th staging system both provided positive net benefits relative to the default “treat-all” and “treat-none” approaches in both training and validation datasets. The net benefit curves for predictions based solely on AJCC stage nearly overlapped with those of the nomogram in the low risk and very high risk threshold regions. However, in the clinical and high risk regions, the nomogram consistently demonstrated a higher net benefit compared to predictions using AJCC staging alone. The average net benefit exceeded 1%, with the maximum net benefit exceeding 2% in all cases compared with AJCC stage. For 1-year survival prediction, the greatest net benefit occurred at the clinical threshold, whereas for 3-year and 5-year survival predictions, the maximum net benefit was observed in the high-risk region.

### Survival analysis for treatment

We compared the differences in KM curves between the imputed and complete-case datasets. Neither the curve shapes nor the log-rank test results showed significant changes. Since the complete-case dataset included a greater variety of pathological subtypes (e.g., NEC, ONB), it was used for the subsequent analysis. The proportional hazards assumption was verified.

**Fig. 5:**
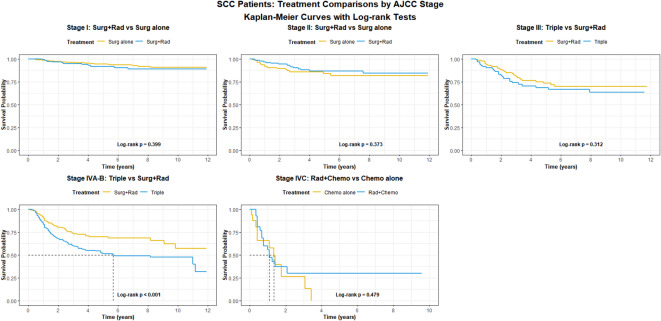
Kaplan-Meier survival analysis for SCC.

The treatment recommendations for SCC varied by AJCC stage. We compared survival outcomes among common and recommended treatment strategies according to stage. The comparison groups were as follows: for stages I and II, surgery alone was compared with surgery+radiation (stage I: 537 vs. 167 patients; stage II: 118 vs. 137 patients); for stages III-IVB, trimodality (surgery + radiation + chemotherapy) was compared with surgery+radiation (stage III: 91 vs. 148 patients; stages IVA-B: 341 vs. 238 patients); and for stage IVC, chemotherapy alone was compared with chemotherapy + radiation (stage IVC: 30 vs. 17 patients). The results are presented in Fig. 5; Table 2.

**Table 2:** The simplified treatment distribution of other six histology types.

Hist	AJCC	Surg alone	Surg + Rad	Rad+Chemo	Trimodality	Total of all patient of each stage
AC	I	66 (61.1%)^1^	23 (21.3%)	0 (0%)	7 (6.5%)	108
AC	II	27 (56.2%)	9 (18.8%)	1 (2.1%)	8 (16.7%)	48
AC	III	13 (31.7%)	17 (41.5%)	0 (0%)	7 (17.1%)	41
AC	IVA	7 (15.9%)	13 (29.5%)	3 (6.8%)	16 (36.4%)	44
AC	IVB	3 (6.1%)	11 (22.4%)	8 (16.3%)	16 (32.7%)	49
AC	IVC	1 (5.9%)	4 (23.5%)	6 (35.3%)	0 (0%)	17
AC	NA	14 (37.8%)	5 (13.5%)	2 (5.4%)	2 (5.4%)	37
ACC	I	15 (35.7%)	21 (50%)	0 (0%)	4 (9.5%)	42
ACC	II	5 (13.5%)	26 (70.3%)	1 (2.7%)	2 (5.4%)	37
ACC	III	15 (24.6%)	30 (49.2%)	1 (1.6%)	9 (14.8%)	61
ACC	IVA	6 (8.8%)	42 (61.8%)	3 (4.4%)	9 (13.2%)	68
ACC	IVB	12 (14.1%)	23 (27.1%)	12 (14.1%)	18 (21.2%)	85
ACC	IVC	2 (9.1%)	4 (18.2%)	6 (27.3%)	4 (18.2%)	22
ACC	NA	4 (15.4%)	10 (38.5%)	2 (7.7%)	0 (0%)	26
UC	I	1 (11.1%)	2 (22.2%)	1 (11.1%)	4 (44.4%)	9
UC	II	0 (0%)	2 (40%)	1 (20%)	2 (40%)	5
UC	III	1 (4.3%)	4 (17.4%)	3 (13%)	13 (56.5%)	23
UC	IVA	3 (6.4%)	0 (0%)	10 (21.3%)	31 (66%)	47
UC	IVB	2 (2.1%)	7 (7.4%)	29 (30.5%)	40 (42.1%)	95
UC	IVC^4^	1 (4.8%)	0 (0%)	10(47.6%)	4 (19%)	21
UC	NA	0 (0%)	0 (0%)	2 (22.2%)	4 (44.4%)	9
PMM	III	86 (33.3%)	146 (56.6%)	1 (0.4%)	11 (4.3%)	258
PMM	IVA	22 (16.7%)	74 (56.1%)	3 (2.3%)	4 (3%)	132
PMM	IVB	9 (16.1%)	27 (48.2%)	0 (0%)	8 (14.3%)	56
PMM	IVC	18 (29%)	7 (11.3%)	0 (0%)	6 (9.7%)	62
PMM	NA	17 (32.1%)	18 (34%)	0 (0%)	3 (5.7%)	53
SCC	I	537(62.8%)	167 (19.5%)	15 (1.8%)	16 (1.9%)	855
SCC	II	118(34.5%)	137 (40.1%)	13 (3.8%)	34 (9.9%)	342
SCC	III	67 (16.5%)	148 (36.5%)	37 (9.1%)	91(22.4%)	406
SCC	IV A-B	125(10.4%)	238 (19.8%)	237(19.8%)	341(28.4%)	1200
SCC	IVC^3^	6 (4.4%)	12 (8.8%)	30 (22.1%)	21 (15.4%)	136
SCC	NA	77 (29.8%)	22 (8.5%)	10 (3.9%)	12 (4.7%)	258
NEC^2^	I	2 (22.2%)	1 (11.1%)	1 (11.1%)	2 (22.2%)	9
NEC	II	0 (0%)	1 (25%)	1 (25%)	2 (50%)	4
NEC	III	0 (0%)	0 (0%)	2 (40%)	3 (60%)	5
NEC	IVA	1 (4.8%)	1 (4.8%)	5 (23.8%)	8 (38.1%)	21
NEC	IVB	0 (0%)	0 (0%)	9 (37.5%)	12 (50%)	24
NEC	IVC	1 (16.7%)	0 (0%)	1 (16.7%)	1 (16.7%)	6
NEC	NA	3 (15%)	2 (10%)	6 (30%)	4 (20%)	20
ONB^4^	NA	113 (23%)	198 (40.3%)	28 (5.7%)	104(21.2%)	491

^1^Percentages indicate the proportion of patients within each stage who received the specific treatment. ^2^Owing to an insufficient sample size for imputation, NEC was reclassified as “Other” in the histology variable during model construction. ^3^17 cases receive chemotherapy alone. ^4^ONB cases were excluded from modeling as all staging data were missing not at random due to the inapplicability of AJCC staging. However, data for both NEC and ONB were retained for descriptive survival analysis, and their treatment details are presented in Table 2.

For stages I and II, no significant difference in survival was observed between surgery alone and surgery+radiation (*P* = 0.399 and 0.373, respectively). Similarly, for stage III SCC, no significant survival difference was found between trimodality and surgery+radiation. In the stage IVA-B cohort, however, a significant difference in survival was noted between trimodality and surgery+radiation (*P* < 0.001). In stage IVC, the limited sample size precluded the detection of a significant survival difference between chemotherapy alone and chemotherapy plus radiation (*P* = 0.479).

**Fig. 6:**
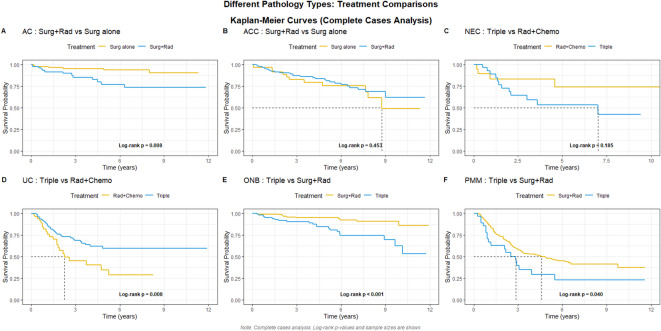
Kaplan-Meier survival analysis of six types of histology.

For the remaining six pathological types, survival outcomes were compared based on their respective recommended and debated treatment approaches, as detailed in Fig. 6; Table 2. In AC, surgery alone was associated with significantly longer survival compared with surgery + radiation (*P* = 0.008; 117 vs. 77 patients). In ACC, no significant survival difference was observed between surgery alone (55 patients) and surgery+radiation (146 patients). In UC, trimodality was associated with a significant survival difference relative to radiation + chemotherapy (*P* = 0.008, 94 vs. 54 patients). In PMM, surgery+radiation was associated with longer survival compared with trimodality (*P* = 0.040, 254 vs. 29 patients,). In NEC, no significant survival difference was found between radiation + chemotherapy (19 patients) and trimodality (28 patients). In ONB, surgery+radiation was associated with a significantly different survival outcome compared with trimodality (*P* < 0.001, 198 vs.104 patients).

## Discussion

The discussion bellow would be divided into two parts: One was about the nomogram and the predictors. The other was the survival analysis of seven histology types.

### The analysis of predictors

We developed the nomogram designed for 6 kinds of rare sinonasal malignancies (AC, ACC, SCC, UC, PMM, Other). This was in contrast to previously published models, which were each limited to a single pathological type, such as AC^7^, SCC^8^, or PMM^10^. While separate validation for each individual subtype was not conducted, this integrated multi-histology model held significant clinical importance, as it provided the available risk stratification tool to assist physicians in managing these uncommon histologies. In clinical decision-making, the value of a general tool lied in its accessibility and broad applicability.

Compared to previously published models, ours diverged in the selection of predictors. Specifically, grade and chemotherapy were not included. Among the three previous models, only the SCC model incorporated chemotherapy.

Our nomogram had a good C-index, which can reach 0.77 in the validation set, indicating that the nomogram has good predictive accuracy. Our nomogram demonstrated a moderate C-index, which was surpassed only by the SNAC model (C-index: 0.80). The reported C-indices for SCC and PMM models were 0.75 and 0.71, respectively. A direct comparison of calibration curves with prior studies was not feasible, as neither the slope/intercept information nor the binning strategy was reported. For our model, the calibration curve performed best for 1-year survival prediction, with a slope of 0.90 in the validation set; however, performance declined for 3- and 5-year predictions, with slopes decreasing to 0.80 and 0.77, respectively. DCA showed a consistent net benefit over the “treat-all” and “treat-none” strategies across most clinical thresholds. Our DCA results were comparable to those of the PMM model, and both demonstrated superior clinical utility compared to using AJCC staging alone. In terms of methodology, firstly, our utilization of the largest sample size to date for model construction, and secondly, our application of data imputation—a strategy not employed in any of the earlier models.

In terms of sex, the number of male patients was significantly higher than that of female patients. It was consistent with the findings of several previous studies^10–13^. Besides, in two other studies, it was found that epithelial sinus cancer (SNC) and sinus adenocarcinoma might be related to exposure to wood dust or leather dust. The populations exposed to these substances were predominantly male^14,15^. Our results indicated that gender was a prognostic factor for survival. Male had a worse survival prognosis compared with female (TR: 0.84, 95%CI: 0.72–0.97). An earlier study also showed that male patients with cancer of the nasal cavity had a worse prognosis^9^. The predictor “Marriage” was also noteworthy. The TR for “Unmarried” was 0.74 (95% CI: 0.63–0.86). In the three other previous models, the direction of risk was consistent. all these results suggested that married patients had a better prognosis. Moreover, beyond this disease, similar patterns have been observed in other types of cancers, such as nasopharyngeal carcinoma^16^ and thyroid carcinoma^17^. We considered that this might be related to family care as well as psychological and financial support.

Unlike previous nomogram constructions, we modified the approach by presenting age as a continuous variable in the nomogram. The TR for age was 0.973 (95% CI: 0.97–0.98). It meaned survival time decreased by approximately 2.7% for each 1 year increase in age. This result was consistent with those of previous models, which also indicated that older age was associated with poorer survival prognosis. In prior research, They had categorized the age groups of 50–64 and 60–69 as medium risk and the reason for the categories were unknown^7,8^.

In terms of AJCC stage and site, the TR for stages IVA, IVB, and IVC were all relatively low, at 0.09 (95% CI: 0.07–0.13), 0.06 (95% CI: 0.04–0.08), and 0.03 (95% CI: 0.02–0.05), respectively. Even for stage II and III, the TR values were 0.29 and 0.14, respectively. Our findings were consistent with previous models, both indicating that advanced stage is an adverse prognostic factor for sinonasal malignancies. Our study directly estimated the extent of survival time reduction using the AFT model. It provided a more intuitive quantitative basis for clinical decision-making. A study based on the SEER database from 1973 to 2011 showed that the site of sinonasal malignancies was predominantly the nasal cavity, with the frontal sinus being the least common site. The most common site, nasal cavity, also had the best prognosis^10^. Our results also indicated that the nasal cavity had the highest proportion of cases (3,386, 58.4%). The nomogram also showed that the nasal cavity had the best survival prognosis (TR: 1.85, 95%CI: 1.44–2.39) compared to the ethmoid and maxillary sinuses.

In terms of radiation, it demonstrated statistical significance in multivariate regression analysis (TR: 2.17, 95% CI: 1.85–2.54). This variable demonstrated a significant correlation with prolonged survival time. Studies also indicated that radiation was associated with better survival outcomes^18,19^. It was often used as a postoperative treatment. A previous observational study also reported an association between the combination of surgery and radiation and improved survival in these patients. Early postoperative intervention might be associated with overall survival^20^.

Chemotherapy was excluded as a predictor. Among the three previously constructed models, only SCC prognosis model had incorporated chemotherapy as a predictive factor. In the latest treatment recommendations, It was only applied in locally advanced such as induction chemotherapy (IC)^21^. Previous meta-analyses indicated that the potential form of chemotherapy was IC^22^. IC had a protective effect on patients with orbital invasion^23^. For patients with unresectable SNSCC (T3-T4 stage), a retrospective study of 127 patients showed that neoadjuvant chemotherapy (NAC) was a viable option because it could provide an opportunity for radical surgery^24^. The combination of radiation and chemotherapy remained the preferred treatment for rarer pathological type such as nasal natural killer cell/T-cell lymphoma (ENKTCL)^21^. A retrospective study of 42 patients with advanced sinonasal malignancies (T3-4NXM0) indicated that patients with a good response to IC might have a better survival rate^25^.

In terms of surgery, it was the most frequently employed treatment modality^26^. In our study, surgery demonstrated the highest TR value (3.26, 95% CI: 2.76–3.77) as a factor associated with improved prognosis. This finding was consistent with the results from the three aforementioned models. However, due to factors such as tumor size and site of invasion, the impact of surgery on prognosis largely depended on whether negative margins could be achieved^21^. A retrospective study of 340 patients found that surgery was associated with improved OS in patients without lymph node metastasis, whereas it had no association with such benefit in those with metastasis^27^. Either open or endoscopic surgery was needed depending on which could fully resect the tumor and there was no difference in survival outcomes between the two^28^. Endoscopic surgery might lower risk of complications compared with open one. A retrospective study of 96 cases involving different pathological types found that extended endoscopic resection was associated with a lower risk of minor complications compared to traditional open surgery for patients with skull base invasion^29^. A prospective, multicenter study involved with different pathological types indicated that the endoscopic approach was associated with better quality of life for patients^30^. Another multicenter study, which included 1,360 patients with transnasal endoscopic surgery showed that different histology was associated with different prognoses^31^.

### The survival analysis of 7 histology types

For SNSCC, no survival difference was observed between those treated with surgery alone and those treated with surgery + radiation in patients with stage I and II in Fig. 5. However, in patients with T2-T4 SNSCC, surgery + radiation was recommended and the control rate could range from 50% to 85%^21,32,33^. In the latest consensus statement, it concluded that radiation should be recommended for locally advanced or poorly differentiated SNSCC^28^. The survival benefit of radiation for patients with stage II has not been confirmed. This result still requires further prospective studies for confirmation. For patients with stage III SNSCC, the survival curves of those receiving trimodality and those treated with surgery+radiation had no significant survival difference. Interestingly, for patients with stage IVA-B SNSCC, surgery+radiation demonstrated a distinct survival advantage compared to trimodality. This observation might be influenced by selection bias, as patients with resectable tumors were more likely to undergo surgery + radiation. For stage IVC patients, the limited sample size precluded a clear comparison between chemotherapy and chemoradiation. In short, while surgery could be clearly established as the primary treatment for stage I, the prognostic value of staging information for guiding treatment selection in other stages remained limited. Ultimately, tumor resectability continued to be the key determinant of prognosis.

For SNAC, evidence-based conclusions regarding treatment strategies remained limited and no prospective studies have compared surgery alone with other treatment methods^21^. The consensus suggested that surgery was recommended for intestinal-type adenocarcinoma (ITAC) and non-ITAC^28^. In Fig. 6**(A)**, Our results showed that surgery alone was associated with a longer survival compared to surgery+radiation. In Table 2, It should be noted that 66 patients (61.1%) were at stage I and 27 patients (56.2%) were at stage II in the patients who received surgery alone. For SNAC, prognosis largely depended on whether the tumor could be completely resected^34^. In the choice of surgical approach for ITAC, no significant difference in postoperative complications was observed between endoscopic and open procedures^35^. Our results showed a favorable prognosis for early-stage SNAC, which may correlate with the efficacy of surgery alone. However, treatment decisions should be based on a comprehensive clinical evaluation and not solely on this prognostic prediction.

For SNACC, although the incidence from 1974 to 2009 was declining with 412 patients, our results showed 353 patients were diagnosed from 2010 to 2021 in the USA^36^. An early study with 105 patients showed that 84% of the patients received surgery + radiation. The 5 year OS was 62.9%. It was better than that of other histology types^37^. Another retrospective study also concluded that SNACC had medium risk of developing local recurrence and distant failure, while SNAC, SNSCC, and PMM had high risk^38^. For the treatment of SNACC, our result showed that surgery + radiation had no statistical significance with surgery alone in Fig. 6**(B)**. Unlike in SNAC, where patients who received surgery were mostly concentrated in stages I-II, those in ACC receiving the surgery+radiation were not concentrated in any specific stage. Instead, they were distributed relatively evenly across all stages except stage IVC. The results of existing studies were few and mostly retrospective for the treatment of ACC. Surgery along with postoperative radiation was recommended^37,39^.

For SNEC, the optimal treatment strategy remained unclear, with ongoing debate regarding the sequencing of surgery, radiation, and chemotherapy^21^. Earlier study suggested that chemotherapy should be administered first, and the subsequent treatment either radiotherapy alone or surgery followed by radiotherapy would be determined based on whether the tumor volume had decreased by more than 50%^40^. In a survival analysis study involving 106 patients, it was also pointed out that definitive chemoradiotherapy or postoperative chemoradiotherapy might be a treatment strategy for SNEC^41^. It had a controversy that whether the treatment strategy for SNEC could be influenced by the degree of differentiation or not^42^. Our results showed that double modality (radiation + chemotherapy, 19 patients) had no statistical significance compared to the trimodality treatment (28 patients) in Fig. 6**(C)**. Due to the limited sample size, it was not possible to determine which patient group achieved a longer survival.

For SNUC, there was a negative result that trimodality was not superior to double modality (surgery+radiation or chemoradiotherapy)^43^. However, our results showed that trimodality was associated with better survival. It also should be noted that those patients with trimodality who were most at stage IV (31 at stage IVA and 40 at stage IVB) in Table 2. Because most patients were T4 stage at diagnosis^44^. A more recent retrospective study also showed that radiation was associated with improved survival of patients with SNUC, regardless of whether it was used preoperatively or postoperatively^18^. Besides radiation, IC was also recommended in recent years^45,46^. The response to IC would determine subsequent treatment: surgery + chemoradiotherapy or only chemoradiotherapy^42,47^. In short, in this study, trimodality therapy was associated with better survival outcomes in SNUC. However, as most patients receiving this regimen had stage IV disease, this association is likely confounded by indication and disease severity, precluding causal inference.

For ONB, our results still indicated that the survival curve for surgery+radiation was associated with better survival outcome compared with trimodality. As shown in Table 2, surgery+radiation was the most commonly chosen treatment option. Although detailed staging information was unavailable, we hypothesized that this survival advantage likely stemmed from differences in disease stage. Given its radiosensitivity and tendency for recurrence, surgery+radiation is a frequently employed combination for ONB, typically indicated for Kadish stage A patients^21^. In contrast, chemotherapy is generally reserved for Kadish stage C patients. However, a study have reported that even in Kadish stage C, the 5 and 10 year OS outcomes between surgery+radiation and trimodality remained similar^48^. In short, our data showed that patients selected for surgery+radiation had better observed survival outcomes. This finding is consistent with established treatment protocols, but as hypothesized, it may largely reflect the selection of earlier-stage (e.g., Kadish stage A) patients for this modality rather than a direct causal effect of the treatment combination.

For PMM, our results showed that surgery+radiation was associated with a better survival outcome with a majority of 146 patients (56.6%) at stage III, and 74 patients (56.1%) at stage IVA. Surgery with negative margin was the initial treatment. Surgery was still the first line therapy. Positive margin was a negative predictor for OS^49^. Radiation might be an option for the treatment^21^. In short, our findings suggested that surgery combined with radiation was associated with improved survival outcomes in this cohort. However, treatment decisions should be individualized based on comprehensive clinical assessment.

## Conclusion

First, we constructed a nomogram to predict the survival prognosis of patients with sinonasal malignancies. The validation set demonstrated good performance, indicating that it can be utilized by clinicians to predict individual survival outcomes and for patient counseling. Second, based on the survival analysis, we provided survival status with common or controversial treatment(s) for 7 histology types of sinonasal malignancies.

## Limitation

For certain rare pathological types, such as ENKTCL, we did not collect data. Therefore, the nomogram was not applicable to predicting the prognosis of the pathological type. Additionally, due to the limitations of the data content, we were unable to determine the specific times and sequences of surgery, radiotherapy, and chemotherapy. As a result, we could not provide more detailed prognostic information.

The high coefficient of variation observed for ACC pathological subtype (CV = 37.1%) suggested considerable variability in the association between ACC and survival outcomes. While the point estimate indicated a 13% increased mortality risk (TR = 1.13, 95% CI: 0.72–1.79), the substantial uncertainty in this estimate limits definitive conclusions regarding the specific prognostic impact of ACC compared to other pathological subtypes. This variability may reflect the relatively small number of ACC cases in our cohort or the inherent heterogeneity within this pathological category.

## Supplementary Information

Below is the link to the electronic supplementary material.

Supplementary Material 1

## Data Availability

The datasets used and/or analysed during the current study are available from the corresponding author on reasonable request.
