# Supplementary material for: Development and validation of a clinico-histological factor-based nomogram for survival in sinonasal malignancies
Source: Sci Rep. 2026 Feb 25;16:11071. doi: 10.1038/s41598-026-41278-9 (PMC13043918; doi:10.1038/s41598-026-41278-9)
Supplement: Supplementary file 1 — Supplementary Material 1 [file 41598_2026_41278_MOESM1_ESM.docx]

Development and Validation of a Clinico-Histological Factor-Based Nomogram for Survival in Sinonasal Malignancies

Chang-Ying Zhong^1^, Chang She^2^, Shuang-Shuang Wang^2,*^

**Table S1.** The histology types of ‘Other’

| **Histology** | **Other**  **(n=348)** |
| --- | --- |
| Nuclear protein in testis (NUT) associated carcinoma | 14 (4.0%) |
| Spindle cell carcinoma, NOS* | 11 (3.1%) |
| Pseudosarcomatous carcinoma | 8 (2.2%) |
| Small cell carcinoma, NOS | 69 (19.8%) |
| Combined small cell carcinoma | 1 (0.3%) |
| Non-small cell carcinoma | 1(0.3%) |
| Papillary carcinoma, NOS | 2 (0.6%) |
| Verrucous carcinoma, NOS | 20 (5.7%) |
| Squamous cell papilloma, inverted, malignant | 2 (0.6%) |
| Lymphoepithelial carcinoma | 6 (1.7%) |
| Basal cell carcinoma, NOS | 7 (2.0%) |
| Basal cell carcinoma, nodular | 2 (0.6%) |
| Adenoid basal cell carcinoma | 1 (0.3%) |
| Schneiderian carcinoma | 10 (3.0%) |
| Basaloid carcinoma | 5 (1.4%) |
| Cribriform carcinoma, NOS | 3 (0.9%) |
| Carcinoid tumor, NOS | 2 (0.6%) |
| Merkel cell carcinoma | 9 (2.6%) |
| Atypical carcinoid tumor | 2 (0.6%) |
| Sclerosing sweat duct carcinoma | 1 (0.3%) |
| Mucoepidermoid carcinoma | 58 (16.7%) |
| Infiltrating duct carcinoma, NOS | 2 (0.6%) |
| Acinar cell carcinoma | 4 (1.1%) |
| Adenosquamous carcinoma | 35 (10.1%) |
| Epithelial-myoepithelial carcinoma | 15 (4.3%) |
| Blue nevus, malignant | 1(0.3%) |
| Sarcoma, NOS | 3 (0.9%) |
| Spindle cell sarcoma | 4 (1.1%) |
| Giant cell sarcoma | 12 (3.4%) |
| Solitary fibrous tumor, malignant | 3 (0.9%) |
| Myxosarcoma | 1 (0.3%) |
| Leiomyosarcoma, NOS | 7 (2.0%) |
| Mixed tumor, malignant, NOS | 3 (0.9%) |
| Carcinoma in pleomorphic adenoma | 3 (0.9%) |
| Carcinosarcoma, NOS | 7 (2.0%) |
| Carcinosarcoma, embryonal | 1 (0.3%) |
| Malignant myoepithelioma | 9 (2.6%) |
| Synovial sarcoma, NOS | 1 (0.3%) |
| Osteosarcoma, NOS | 2 (0.6%) |
| Peripheral neuroectodermal tumor | 1 (0.3%) |

NOS: Not otherwise specified; neuroendocrine carcinoma: 89 cases were in the ‘Other’ when constructing the AFT model.

**Table S2** Comparison of Variables Before and After Imputation

| **Variables** | **Before imputing** | | **After imputing** | | **Percentage in change（%）** |
| --- | --- | --- | --- | --- | --- |
| **Age** | 63.459 | | 63.459 | | 0% |
| **Race** |  | | | | |
| White | 4606 | 79.5% | 4639 | 80.1% | (+0.6%) |
| Black | 572 | 9.9% | 576 | 9.9% | (+0%) |
| Asian | 426 | 7.4% | 429 | 7.4% | (+0%) |
| Other | 150 | 2.6% | 151 | 2.6% | (+0%) |
| **Marriage** |  | | | | |
| Married | 3018 | 52.1% | 3274 | 56.5% | 4.4% |
| Unmarried | 2309 | 39.8% | 2521 | 43.5% | 3.7% |
| **Hist** |  |  |  |  |  |
| AC | 344 | 5.9% | 356 | 6.1% | 0.2% |
| ACC | 341 | 5.9% | 351 | 6.1% | 0.2% |
| Other | 947 | 16.3% | 988 | 17% | 0.7% |
| PMM | 561 | 9.7% | 574 | 9.9% | 0.2% |
| SCC | 3197 | 55.2% | 3305 | 57% | 1.8% |
| UC | 209 | 3.6% | 221 | 3.8% | 0.2% |
| **AJCC** |  | | | | |
| I | 1153 | 19.9% | 1409 | 24.3% | 4.4% |
| II | 486 | 8.4% | 556 | 9.6% | 1.2% |
| III | 897 | 15.5% | 1027 | 17.7% | 2.2% |
| IVA | 1138 | 19.6% | 1312 | 22.6% | 3% |
| IVB | 930 | 16.1% | 1108 | 19.1% | 3.1% |
| IVC | 318 | 5.5% | 384 | 6.6% | 1.1% |

**Table S3** The comparison of TR values before and after imputation.

| **Variable** | **TR**  **(Complete)** | **95% CI**  **(Complete)** | **P**  **(Complete)** | **TR**  **(Imputed)** | **95% CI**  **(Imputed)** | **P**  **(Imputed)** | **TR Difference** | **TR % Difference** |
| --- | --- | --- | --- | --- | --- | --- | --- | --- |
| Age | 0.981 | 95%CI:  0.975, 0.987 | <0.001 | 0.976 | 95%CI: 0.971, 0.981 | <0.001 | -0.005 | 0.50% |
| Race  Black | 1.16 | 95%CI:  0.815, 1.651 | 0.41 | 1.172 | 95%CI: 0.843, 1.63 | 0.345 | 0.012 | 1% |
| Race  Other | 0.953 | 95%CI:  0.561, 1.617 | 0.857 | 1.011 | 95%CI: 0.613, 1.669 | 0.965 | 0.058 | 6.10% |
| Race  White | 1.161 | 95%CI:  0.879, 1.533 | 0.294 | 1.149 | 95%CI: 0.885, 1.491 | 0.296 | -0.012 | 1% |
| Marriage  Unmarried | 0.765 | 95%CI:  0.656, 0.893 | <0.001 | 0.728 | 95%CI: 0.624, 0.849 | <0.001 | -0.037 | 4.80% |
| Hist  ACC | 1.129 | 95%CI:  0.717, 1.779 | 0.6 | 1.1 | 95%CI: 0.697, 1.738 | 0.682 | -0.029 | 2.60% |
| **Hist**  **Other** | **0.677** | **95%CI:**  **0.45, 1.018** | **0.061** | **0.592** | **95%CI: 0.401, 0.873** | **0.008** | **-0.085** | **12.60%** |
| Hist  PMM | 0.299 | 95%CI:  0.199, 0.451 | <0.001 | 0.289 | 95%CI: 0.187, 0.446 | <0.001 | -0.01 | 3.30% |
| Hist  SCC | 0.677 | 95%CI:  0.476, 0.964 | 0.031 | 0.66 | 95%CI: 0.463, 0.941 | 0.022 | -0.017 | 2.50% |
| Hist  UC | 0.514 | 95%CI:  0.317, 0.833 | 0.007 | 0.444 | 95%CI: 0.273, 0.724 | 0.001 | -0.07 | 13.60% |
| Sex  Male | 0.81 | 95%CI:  0.691, 0.95 | 0.009 | 0.836 | 95%CI: 0.72, 0.971 | 0.019 | 0.026 | 3.20% |
| Site  Maxillary sinus | 0.8 | 95%CI:  0.608, 1.053 | 0.111 | 0.837 | 95%CI: 0.647, 1.082 | 0.175 | 0.037 | 4.60% |
| Site  Nasal cavity | 1.722 | 95%CI:  1.308, 2.266 | <0.001 | 1.826 | 95%CI: 1.413, 2.36 | <0.001 | 0.104 | 6% |
| AJCCII | 0.323 | 95%CI:  0.226, 0.461 | <0.001 | 0.286 | 95%CI: 0.202, 0.407 | <0.001 | -0.037 | 11.50% |
| AJCCIII | 0.167 | 95%CI:  0.122, 0.229 | <0.001 | 0.146 | 95%CI: 0.103, 0.207 | <0.001 | -0.021 | 12.60% |
| AJCCIVA | 0.109 | 95%CI:  0.081, 0.147 | <0.001 | 0.099 | 95%CI: 0.071, 0.139 | <0.001 | -0.01 | 9.20% |
| AJCCIVB | 0.065 | 95%CI:  0.047, 0.089 | <0.001 | 0.063 | 95%CI: 0.046, 0.086 | <0.001 | -0.002 | 3.10% |
| AJCCIVC | 0.035 | 95%CI:  0.024, 0.051 | <0.001 | 0.033 | 95%CI: 0.022, 0.05 | <0.001 | -0.002 | 5.70% |
| Surg  Yes | 3.293 | 95%CI:  2.779, 3.903 | <0.001 | 3.17 | 95%CI: 2.713, 3.703 | <0.001 | -0.123 | 3.70% |
| Radiation  Yes | 2.226 | 95%CI:  1.874, 2.644 | <0.001 | 2.268 | 95%CI: 1.924, 2.674 | <0.001 | 0.042 | 1.90% |
| **Chemotherapy**  **Yes** | **0.949** | **95%CI:**  **0.788, 1.142** | **0.579** | **0.837** | **95%CI: 0.697, 1.004** | **0.055** | **-0.112** | **11.80%** |

**Table S4** multivariate interpolation coefficient table

| **Variable** | **Coefficient** | **Std**  **Error** | **CV percent** | **TR** |
| --- | --- | --- | --- | --- |
| Age | 0.02 | 0.00 | 2.60 | 0.98 |
| SexMale | 0.18 | 0.01 | 6.80 | 0.84 |
| MarriageUnmarried | 0.30 | 0.02 | 7.80 | 0.74 |
| HistACC | 0.13 | 0.05 | 37.10 | 1.13 |
| HistOther | 0.54 | 0.05 | 9.90 | 0.58 |
| HistPMM | 1.20 | 0.08 | 6.30 | 0.30 |
| HistSCC | 0.43 | 0.04 | 8.80 | 0.65 |
| HistUC | 0.87 | 0.06 | 7.20 | 0.42 |
| SiteMaxillary sinus | 0.17 | 0.02 | 11.40 | 0.84 |
| SiteNasal cavity | 0.62 | 0.02 | 3.30 | 1.85 |
| AJCCII | 1.25 | 0.05 | 4.30 | 0.29 |
| AJCCIII | 1.96 | 0.08 | 4.20 | 0.14 |
| AJCCIVA | 2.36 | 0.08 | 3.30 | 0.09 |
| AJCCIVB | 2.83 | 0.05 | 1.90 | 0.06 |
| AJCCIVC | 3.46 | 0.09 | 2.50 | 0.03 |
| SurgYes | 1.17 | 0.01 | 0.90 | 3.23 |
| RadiationYes | 0.77 | 0.02 | 2.50 | 2.17 |

CV: Coefficient of variation
